# Peripheral Calcifying Odontogenic Cyst: A rare case report

**DOI:** 10.4317/jced.55137

**Published:** 2018-11-01

**Authors:** Liana-Cristina-Melo-Carneiro Costa, Joaquim-Barbosa-Matias Neto, Eliene-Magda de-Assis, Hayder-Egg Gomes, Tiago-Johnston Leitão, Renzo-Rafael-Cevallos Vasconcelos, Paulo-Eduardo-Alencar Souza, Martinho-Campolina-Rebello Horta

**Affiliations:** 1 Graduate Program in Dentistry, School of Dentistry, Pontifical Catholic University of Minas Gerais (PUC Minas), Belo Horizonte, MG, Brazil; 2 Oral Pathology Section, School of Dentistry, Pontifical Catholic University of Minas Gerais (PUC Minas), Belo Horizonte, MG, Brazil; 3 Faculdade Pitágoras de Ipatinga, Ipatinga, MG, Brasil; 4 Laboratório CIAP, Divinópolis, MG, Brasil; 5 Federal University of Sao Joao del-Rei (UFSJ), Divinópolis, MG, Brasil; 6 Private orthodontics practice, Divinópolis, MG, Brasil

## Abstract

The Calcifying Odontogenic Cyst (COC) is a simple cyst lined by ameloblastoma-like epithelium with ghost cells. The peripheral COC is a rare lesion and few reports have been published considering its clinical and histopathological features. This article aimed to report on a case of a peripheral COC, discussing its clinical, imaginological and histopathological features. A 9-year-old male patient presented a 10x5 mm painless nodule in the palatal mucosa of the left central incisor. Panoramic, occlusal and periapical radiographs did not show alterations. A computed tomography exam showed a slight soft tissue swelling located in the palatal mucosa of the left maxillary central incisor. An excisional biopsy was performed. The histopathological analysis showed a cystic lesion adhered to an oral mucosa fragment and lined by an ameloblastoma-like epithelium with ghost cells. The diagnosis of peripheral COC was established and the patient has been disease-free for 5 years. Although rare, peripheral COC is an important lesion that should be considered as a differential diagnosis of gingival hyperplastic lesions.

** Key words:**Calcifying odontogenic cyst, odontogenic tumors, peripheral calcifying odontogenic cyst.

## Introduction

First described by Gorlin *et al.* in 1962 (1), the Calcifying Odontogenic Cyst (COC) is a simple cyst lined by ameloblastoma-like epithelium with ghost cells (2,3). The 4th edition of the World Health Organization’s Classification of Head and Neck Tumours, published in 2017, renamed and reclassified this lesion as an odontogenic cyst, since it had been categorized in 2005 as an odontogenic tumor (4).

COC is an uncommon lesion, representing 0.1% of all records and 1.3% of all odontogenic cysts in a recent multicentric study evaluating 198,350 histopathological records from oral and maxillofacial pathology services (3).

Although most of the COC are intraosseous, peripheral lesions can also occur. The peripheral COC show the same microscopic features of intraosseous COC, but is located in the soft tissues of tooth-bearing areas (5). It is an even rarer lesion, corresponding to fewer than 3% of the COC (3).

This article aimed to report on a case of a peripheral COC diagnosed in a 9-year-old male, discussing its clinical, imaginological and histopathological features.

## Case Report

A 9-year-old male patient accompanied by his mother sought dental assistance complaining of a palatal swelling. The lesion was painless and had initially been observed 5 months earlier. The medical history was noncontributory.

**Figure 1:**
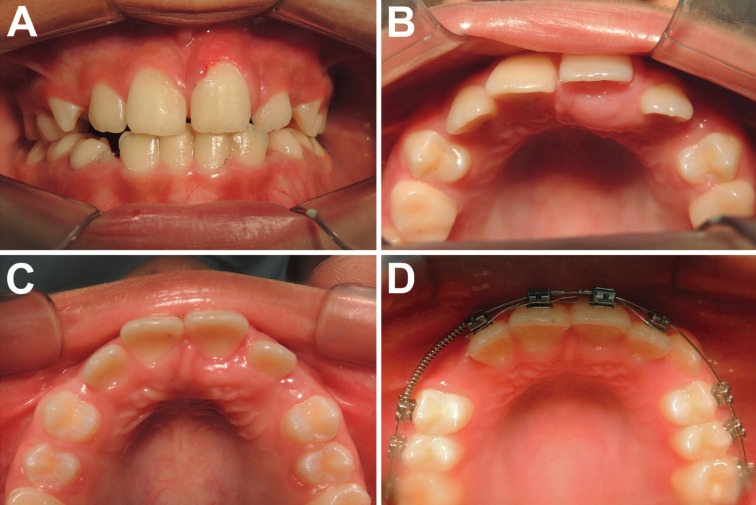
A: Intraoral physical examination; B: Intraoral physical examination showing a 10x5 mm painless nodule in the palatal mucosa of the left central incisor; C: Follow-up visit two months after the excisional biopsy showing adequate healing; D: Follow-up visit one year after the surgical removal showing no recurrence.

The intraoral physical examination revealed a 10x5 mm, ill-delimited, firm and sessile nodule, located in the palatal mucosa of the left central incisor (Fig. 1A,B). The overlying mucosa was intact and normal in color. Buccoversion of the adjacent teeth was observed, but no mobility or other periodontal alterations.

**Figure 2:**
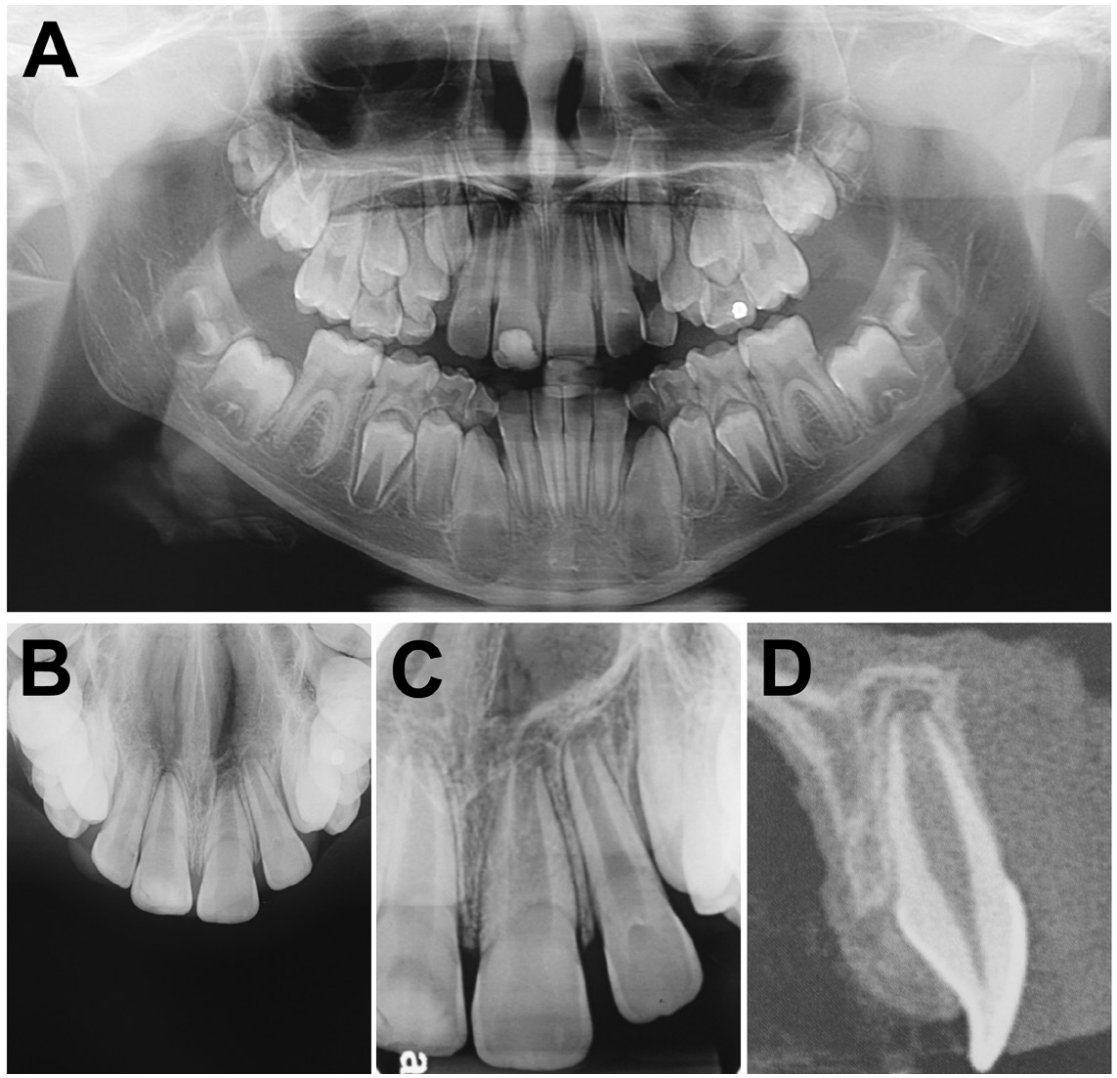
A: Panoramic radiography with no related bone alterations; B: Occlusal radiography with no related bone alterations; C: Periapical radiography with no related bone alterations; D: Cone beam computed tomography (CBCT) exam showing no related bone alterations, but a slight soft tissue swelling in the palatal mucosa of the left maxillary central incisor.

Panoramic, occlusal and periapical radiographs did not show alterations in the area adjacent to the lesion (Fig. 2A-C). A cone beam computed tomography (CBCT) exam also revealed no related bone alterations, but show a slight soft tissue swelling located in the palatal mucosa of the left maxillary central incisor (Fig. 2D).

The main diagnosis hypotheses included fibrous hyperplasia, peripheral ossifying fibroma, pyogenic granuloma and peripheral giant cell granuloma. An excisional biopsy was performed under local anesthesia. During the lesion’s surgical removal, no superficial bone resorption was observed. Grossly, a cyst-like structure was observed adhered to the oral mucosa. The sample was sent to a Pathology Laboratory.

**Figure 3:**
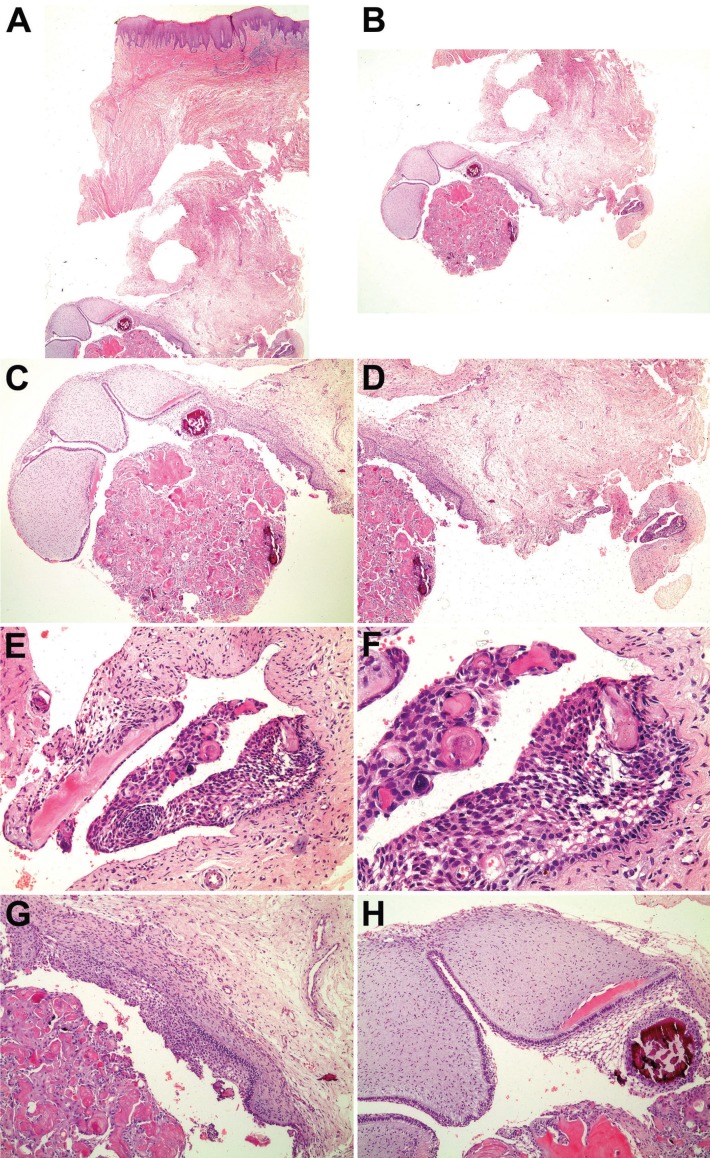
A, B, C, D: Microscopic examination showing an oral mucosa fragment with a cystic lesion adhered to the deeper lamina propria (A: HE x20; B: HE x20; C: HE x40; D: HE x40); E and F: The cystic capsule was lined by an ameloblastoma-like epithelium with ghost cells showing occasional calcification (E: HE x200; F: HE x400); G: Ghost cells were abundantly observed in the cyst lumen (HE x100); H: Soft and hard dental tissue resembling a developing odontoma in the cyst wall (HE x100).

The histological examination revealed an oral mucosa fragment covered by a stratified squamous keratinized epithelium showing areas of hyperplasia (Fig. 3A). A cystic lesion was observed adhered to the deeper lamina propria (Fig. 3A-D). The cystic capsule, formed by dense fibrous connective tissue, was lined by an ameloblastoma-like epithelium (Fig. 3E). The basal layer cells of this lining epithelium were cubic or columnar, showing reversed polarity and hyperchromatic nuclei, disposed in palisade. The cells of the suprabasal layers were sometimes loosely arranged. Enlarged epithelial cells with eosinophilic cytoplasm and no nucleus (ghost cells) were sporadically found in the lining epithelium (Fig. 3E,F) and abundantly observed in the cyst lumen (Fig. 3C,G). Calcification of ghost cells was also observed (Fig. 3C-F). Juxtaepithelial dentinoid could be seen in the cyst capsule (Fig. 3E). Moreover, soft and hard dental tissue resembling a developing odontoma were observed in the cyst wall (Fig. 3C,H).

The diagnosis of peripheral COC was established. In a follow-up visit two months after the excisional biopsy, the area was adequately healed (Fig. 1C). One year after the surgical removal, no recurrence was observed (Fig. 1D). The patient has been disease-free for 5 years. Informed consent has been obtained by the patient.

## Discussion

The peripheral COC is a rare lesion and there are few studies in the literature concerning its clinical and histopathological features. In 1991, Buchner *et al.* (5) revised the 38 well-documented cases published in the English-language literature and reported additional 7 lesions, totalizing 45 cases. Afterward, Resende *et al.* (6) reviewed all the cases reported by Buchner *et al.* (5) and the succeeding reports from 1991 to 2010, finding 44 acceptable cases. Finally, Chrcanovic and Gomez (7) in a systematic review employing consistent eligibility criteria, found a total of 55 cases reported until 2016.

Peripheral COC can affect patients in a wide age range, with a mean of 41.8 years. There is a slight predilection for women, in which the lesion appears in a statistically higher age (49.1 years) when compared to men (33.3 years) (7). The case reported occurred in a 9-year-old male patient, much younger than this average age. In fact, only one of the 45 peripheral COC reported by Buchner *et al.* (5) affected patients between 0 and 9 years old.

Most of the reported peripheral COC developed as painless, well-delimited and smooth-surface nodules on the gingiva or alveolar mucosa (5,6). The lesion size varies from 0.5 to 3.0 cm (7). The majority of the cases affects the mandible and the incisor-canine area is the most common location (7). The reported case presented almost all these clinical features. As described in the present case, the most common clinical differential diagnosis are common gingival hyperplastic lesions (5,6).

The histopathological analysis of the reported case disclosed typical findings of COC such as the fibrous connective capsule lined by an ameloblastoma-like epithelium and the presence of ghost cells, as well as areas of dentinoid formation in the capsule (8-10). Moreover, soft and hard dental tissue resembling a developing odontoma were observed in the cyst wall, similar to one of the cases reported by Buchner *et al.* (5).

Conservative surgical excision is the standard treatment for peripheral COC and recurrences are rare (5-7). This surgical approach was effective in the case reported, with no signs of recurrence after a 5-year follow-up.

Although rare, peripheral COC is an important lesion that should be considered as a differential diagnosis of gingival hyperplastic lesions.
